# Effects of Chronic Heat Stress on Kidney Damage, Apoptosis, Inflammation, and Heat Shock Proteins of Siberian Sturgeon (*Acipenser baerii*)

**DOI:** 10.3390/ani13233733

**Published:** 2023-12-02

**Authors:** Zhaoxin Jing, Qianyu Chen, Chaozhan Yan, Chaoyang Zhang, Zihan Xu, Xiaoli Huang, Jiayun Wu, Yunkun Li, Shiyong Yang

**Affiliations:** 1 College of Animal Science and Technology, Sichuan Agricultural University, Chengdu 611130, China; 13208112878@163.com (Z.J.); dkycqy@163.com (Q.C.); hxldyq@126.com (X.H.); 2 College of Life Sciences, Sichuan Agricultural University, Ya’an 625000, Chinaabc2004wjy@163.com (J.W.); yunkun.li@sicau.edu.cn (Y.L.)

**Keywords:** *Acipenser baerii*, heat stress, kidney tissue, heat shock proteins

## Abstract

**Simple Summary:**

Heat is a common stressor in aquatic environments and can cause damage to organs such as the kidneys, which in turn affects normal physiological functions in aquatic animals. The effects of heat stress on the kidney of Siberian Sturgeon (*Acipenser baerii*) were investigated using histologic observation, flow cytometry assay, and RT-qPCR technique. Heat stress caused damage to the kidneys, leading to inflammatory cell infiltration, a significant increase in apoptosis as well as a significant increase in plasma creatinine, and induced significant expression of the heat shock protein *GRP75* mRNA. However, the mRNA expression levels of inflammatory factors (*IL-1β* and *TNF-α*) and heat shock protein (*HSP70* and *HSP90*) did not change significantly.

**Abstract:**

Chronic heat stress caused by global warming can have serious implications for fish survival. The kidney plays a central role in many homeostatic functions, including water and electrolyte regulation. However, there is limited knowledge about the effect of heat stress on fish kidneys. In this study, water temperatures were increased from 20 °C to 24 °C and 28 °C in 8 days at a warming rate of 1 °C/d, and then maintained for 12 days. We investigated the effects of mild heat stress (24 °C) and high heat stress (28 °C) on Siberian Sturgeon (*Acipenser baerii*) kidneys using histological observation, flow cytometry detection, and RT-qPCR. Our histological observations revealed that heat stress caused significant infiltration of inflammatory cells in the kidney, especially at 28 °C. The flow cytometry assay demonstrated a significant increase in the number of apoptotic cells after heat stress at 28 °C compared to a control group at 20 °C (*p* = 0.033). The level of plasma creatinine was significantly increased in the 28 °C group compared to the control group (*p* = 0.001). In addition, the mRNA expression levels of heat shock protein *GRP75* increased (*p* = 0.009). The results indicate that heat stress at 28 °C caused damage to the kidneys of *A. baerii* and triggered the protective response of heat shock proteins. In conclusion, this study contributes to the understanding of the coping strategies of the kidney of *A. baerii* for chronic heat stress.

## 1. Introduction

Since the 1850s, global temperatures have continued to rise, triggering profound changes in environmental conditions, including water temperatures that are critical to the survival of aquatic animals. Due to global warming, heat stress poses a serious threat to the behavior, growth, development, reproduction, and even death of aquatic animals [1,2,3,4]. Consequently, climate-induced heat stress is increasingly considered to be a worrisome issue [5]. Although the defense system of fish can resist the adverse effects of heat stress to some extent [6], surpassing their temperature tolerance can induce important organismal changes such as protein misfolding and apoptosis [7,8]. Apoptosis is a physiological protective mechanism that involves the activation, expression, and regulation of a range of genes to remove excess, damaged, or dangerous cells from the body [9,10]. However, severe apoptosis can also lead to tissue and organ damage and abnormal function [11]. Although heat stress has been shown to induce apoptosis in fish such as *Prochilodus lineatus* [12], *Paralichthys olivaceus* [13], and *Takifugu obscurus* [14], there are few reports on the effects of heat-stress-induced apoptosis in sturgeon.

Fish are ectothermic animals, and drastic changes in water temperature can seriously affect the production of innate immunity [15]. A variety of cytokines involved in the immune response of fish vary with water temperature in response to ambient temperature [16]. Thus, changes in cytokine expression levels can represent changes in innate immune function in fish [17]. It has been found that heat stress severely affects the innate immune response of *Micropterus salmoides* by down-regulating the expression of pro-inflammatory cytokines while exacerbating the inhibitory effects on the complement and coagulation systems, thus weakening the resistance of *Micropterus salmoides* to pathogen infection [18]. Heat stress leads to overproduction of pro-inflammatory factors, resulting in damage to several tissues of the fish, such as gills, liver, and spleen [4]. Previous studies in our laboratory found that heat stress at 24 °C and 28 °C caused severe damage to the gills and intestine of *A. baerii*. The epithelial cells of gill filaments degenerated and hyperostosis occurred after heat stress at 24 °C and 28 °C, particularly at 28 °C where necrosis appeared [19]. Similarly, mucosal epithelial cells showed marked signs of necrosis after heat stress at 24 °C and 28 °C, and shedding cells were seen in the lumen of the valve intestine accompanied by a large amount of inflammatory cell infiltration [20]. The fish kidney is a complex organ with complex structure and diverse functions, which not only can maintain the water-salt balance of the fish body and regulate its osmotic pressure, but also has immune, hematopoietic, and excretory functions [21,22,23]. It plays an important role in the process of resistance to invasion of foreign pathogenic bacteria and unfavorable external stress [24,25]. It was found that heat stress at 24 °C resulted in the high induction of many genes involved in maintaining homeostasis in the body or adapting to stress and stimuli at high temperatures in *Oncorhynchus mykiss* head kidney tissue [26]. In addition, miRNA sequencing of *Oncorhynchus mykiss* head kidney tissues revealed that heat stress at 24 °C resulted in significant changes in regulatory pathways such as protein processing in the endoplasmic reticulum, NOD-like receptor signaling pathway, and phagosomes [27]. However, relatively few studies have examined the effects of heat stress on the kidney of sturgeon. High water temperatures also trigger protein denaturation, which can be rescued by heat shock proteins (HSPs), thus repairing damaged proteins and ensuring normal protein synthesis [28]. Previous studies have found changes in mRNA expression levels of heat shock protein genes and immune-related genes to counteract heat stress [29,30]. It was found that heat stress at 37 °C activated the expression of *ERK1/2* and *HSF1* mRNA in *Scophthalmus maximus* kidney cells and induced the expression of *HSP90* mRNA in kidney cells to protect against heat stress [31]. However, the response of HSPs to heat stress in sturgeon kidneys is not clear.

Sturgeon, as a living fossil, is one of the oldest species in the class *Actinopterygii*, widely distributed throughout the Northern Hemisphere [32]. Because of the high nutritional value of caviar, an artificial culture of sturgeon to obtain caviar has become one of the main economic fish in Chinese specialty aquaculture [33]. There are more than 13 species of sturgeon or their hybrids in China, with *Acipenser baerii* (*A. baerii*) being one of the main cultured species [34,35]. The optimum living temperature of *A. baerii* is about 20 °C. It has been proven that warm (24 °C) or overheated water (28 °C) can cause a stress reaction in *A. baerii*, leading to tissue damage and dysfunction in the gills, skin, intestines, liver, and spleen, and even leading to death. Currently, research on *A. baerii* focuses on breeding, growth performance, disease resistance, and germplasm identification, but very little research has been done on the effects of increased water temperatures due to global warming on *A. baerii*, especially on the kidneys. Thus, whether heat stress poses a threat to the kidneys of sturgeon remains to be explored.

In this study, we evaluate the effects of heat stress on kidney histopathology, apoptosis, inflammation, and HSPs. We examined the expression of key HSP-related genes (*HSP70*, *HSP90*, and *GRP75*) and innate immunity-related genes (*IL-1β* and *TNF-α*). Our objective is to explore the effects of heat stress on kidney structure, function, apoptosis, immune response, and heat shock proteins in *A. baerii*.

## 2. Materials and Methods

### 2.1. Fish Maintenance and Treatment Protocols

All *A. baerii* used in this study were collected from Sichuan Runzhao Fisheries Co., Ltd. (Chengdu, China), and were randomly sampled from the same pond (temperature = 15.0 ± 0.3 °C, pH = 6.9 ± 0.16). A total of 180 fish were used for the experiment, with an average body length of 23.5 ± 2.41 cm and weight of 76.4 ± 9.45 g. The fish were fed commercial feed (Haida Group Co., Ltd., Jianyang, China) at a rate of 1% of their body weight three times per day (at 8:00 am, 14:00 pm, and 20:00 pm) and were acclimated in the tanks (40 cm × 80 cm × 50 cm) at 20 °C for two weeks prior to the experiment. The fish were then randomly assigned to one of three groups: a control group kept at 20 °C and two elevated temperature groups at 24 °C and 28 °C.

**Figure 1:**
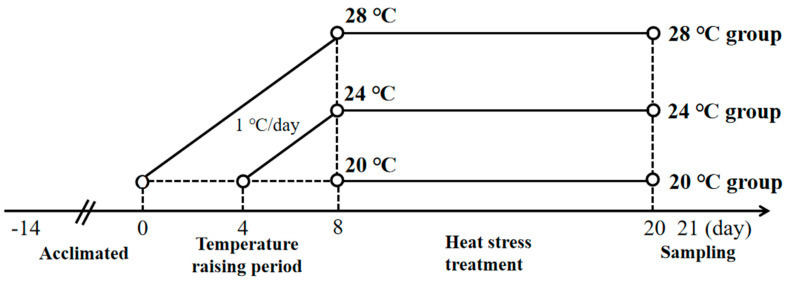
Experimental treatment protocols. Temperature control Day −14 to 0: Temperature was controlled at 20 °C with the heating rods during acclimatization. Day 1 to 8: Temperature was raised to 24 °C and 28 °C with the heating rods at 1 °C/d. Day 9 to 20: the temperature of the three groups was maintained at 20 °C, 24 °C and 28 °C, respectively, using the heating rods. Day 21: Heating ended, and samples collected. The group with a pre-sampling temperature of 20 °C is the 20 °C group. The group with a pre-sampling temperature of 24 °C is the 24 °C group. The group with a pre-sampling temperature of 28 °C is the 28 °C group.

Each group consisted of four parallel tanks (40 cm × 80 cm × 50 cm) with 15 fish each. The experimental heating and heat treatment steps are illustrated in Figure 1. During the two weeks of domestication, the water temperature was maintained at 20 ± 0.5 °C using heating rods (Hangzhou Gerson Trading Co., Ltd., Hangzhou, China). Subsequently, the water temperature was increased by 1 °C/day using heating rods until the experimental temperatures of 24 °C and 28 °C were both reached on the 8th day, and then maintained until the 20th day. The specific heating scheme is described in detail in our previous study [20]. We placed two heating rods in each tank (40 cm × 80 cm × 50 cm) to maintain or raise the temperature. During the treatment period, oxygen was continuously supplied via oxygen pumps for 24 h. On day 21, the fish in each group were euthanized using tricaine mesylate (MS-222) (Sigma-Aldrich, Beijing, China). We poured MS-222 into a round basin (0.3 m diameter and 0.2 m height), added water to a concentration of 35 ppm, and put the fish into the basin to anesthetize them for 5–10 min after the end of heating, and then fished them out after their equilibrium was out of balance and they went into deep anesthesia. Subsequently, the fish were dissected, blood was collected with a sodium heparin tube and plasma creatinine levels were measured, and their kidney tissues were examined for histopathology, apoptosis, immune-related genes, and heat shock protein-related genes.

### 2.2. Examination of Histopathology

On day 21, the fish were euthanized, and kidney tissues were collected from three fish in each group for histological examination. Tissue specimens were fixed in 4% paraformaldehyde solution for 3 d. The fixed kidney tissues were taken out and rinsed with 70% ethanol three times, respectively, and then gradient dehydration was carried out using the concentrations of 70%, 80%, 95%I, 95%II, anhydrous ethanol I, and anhydrous ethanol II for 1 min each, sequentially. After dehydration, the transparency was carried out sequentially using xylene I and xylene II, each for 40 min. Wax dipping was carried out using wax cup I for 40 min, wax cup II for 50 min, and wax cup III for 1 h, and then embedded. The wax blocks were sliced into 5 μm sections and placed on slides and baked at 50 °C for 30 min, and then soaked in xylene I and xylene II for 15 min each for dewaxing. The sections were sequentially stained with hematoxylin phosphotungstate for 30 min, 5% glacial acetic acid for 40 s, and eosin for 5 min. Gradient dehydration was carried out sequentially with concentrations of 70%, 80%, 95%I, 95%II, anhydrous ethanol I, and anhydrous ethanol II for 1 h each over a period of time. Xylene I and Xylene II were used sequentially for 40 min each for transparency. After neutral sealing with glue, the tissue sections were examined under a light microscope (Nikon, Tokyo, Japan).

### 2.3. Flow Cytometry Detection of Apoptosis

Kidney tissues from three fish in each group were collected and fixed separately in pre-cooled 1× PBS solution for subsequent detection of apoptosis. The kidney was incubated with Annexin V-FITC/PI (Thermo Fisher, Waltham, MA, USA) for 25 min at room temperature, and the percentage of apoptotic cells was measured using a flow cytometer (Backman, Pasadena, CA, USA). FITC was excited at a wavelength of 488 nm and emitted at 525 nm, while PI was excited at a wavelength of 535 nm and emitted at 615 nm.

For the flow cytometry detection of apoptosis, a single-cell suspension of 100 μL at a concentration of 1 × 10^6^ cells/mL was taken, centrifuged at 300× *g* for 5 min, and the supernatant was discarded. The cells were then resuspended in 200 μL of freshly prepared 1× binding buffer. Annexin V-FITC staining fluorescent dye (5 μL) was added, and the cells were stained for 10 min at room temperature while being protected from light. Next, PI staining (10 μL) was added and incubated for 5 min at room temperature, avoiding light. Finally, 400 μL of PBS was added, and the cells were resuspended and immediately assayed on the machine. The data were analyzed using Kaluza 2.1 software after being detected by a CytoFLEX flow cytometer (Backman, Pasadena, CA, USA).

### 2.4. Plasma Creatinine Test

The creatinine assay kit (Nanjing Jianjian, Hangzhou, China) was used to detect creatinine levels in plasma according to the instructions. A 96-well flat-bottomed enzyme labeling plate (Nanjing Jianjian, Hangzhou, China) was prepared and divided into assay group (T), standard group (S), and blank group (B), and three replicates were set for each sample in the group. Next, 6 μL of individual plasma samples under different treatments, standards, and double-distilled water were added to groups T, S, and B, respectively. 180 μL of enzyme solution A was added to each group, incubated at 37 °C for 5 min, and the absorbance value was measured at 546 nm. Subsequently, 60 μL of enzyme solution B was added to each group, incubated at 37 °C for 5 min, and the absorbance values were measured at 546 nm. Finally, creatinine content (μmol/L) was calculated according to the formula. All the operations strictly followed the manufacturer’s instructions.

### 2.5. Real-Time Quantitative PCR

Kidney tissues were collected from five fish in each group and stored separately in −80 °C for subsequent mRNA detection. Total RNA was extracted from kidney tissue using animal total RNA isolation kits (Foregene, Chengdu, China), following the manufacturer’s instructions. The integrity of the total RNA was evaluated using 1.0% agarose gel electrophoresis, while the purity and concentration of the total RNA were measured using a NanoDrop spectrophotometer (Thermo Fisher, Waltham, MA, USA). Each sample’s total RNA was reverse-transcribed with oligo-dT primers using a PrimeScript RT reagent kit with gDNA Eraser (TaKaRa, Dalian, China), and subsequently stored at −20 °C until analysis.

The CFX96^®^ Real-Time System (Bio-Rad, Hercules, CA, USA) was used for quantitative real-time PCR. The amplification reaction mixture volume was 10 μL, consisting of 5 μL of 2× Real PCR EasyTM Mix-SYBR (Foregene, Chengdu, China), 1 μL of the cDNA sample, 0.5 μL each of 10 μmol/L forward and reverse primers, and 3 μL of RNase-free water.

Gene-specific primers for the tumor necrosis factor-α (*TNF-α*), interleukin-1β (*IL-1β*), heat shock protein 90 (*HSP90*), heat shock protein 70 (*HSP70*), and glucose regulated protein 75 (*GRP75*) genes were designed using Primer Premier 5.0 software (Table 1). The amplification protocol included an initial pre-denaturation step at 95 °C for 1 min, followed by 40 cycles of denaturation at 95 °C for 10 s, annealing at the corresponding temperature for 30 s, and dissociation curve analysis (melting curve) to verify the amplification of a single product. Each sample was run in triplicate. The relative expression ratio of target genes was calculated using the 2^−ΔΔCt^ method and normalized with the expression of the reference gene *β-actin*.

### 2.6. Statistical Analysis

The data are presented as mean ± SEM. Statistical analysis was conducted using SPSS version 22.0 software, employing one-way ANOVA. To assess the differences between the groups, Least Significant Difference (LSD) was utilized. * *p* < 0.05, ** *p* < 0.01.

## 3. Results

### 3.1. Histopathological Changes in Kidney Tissue under Heat Stress

**Figure 2:**
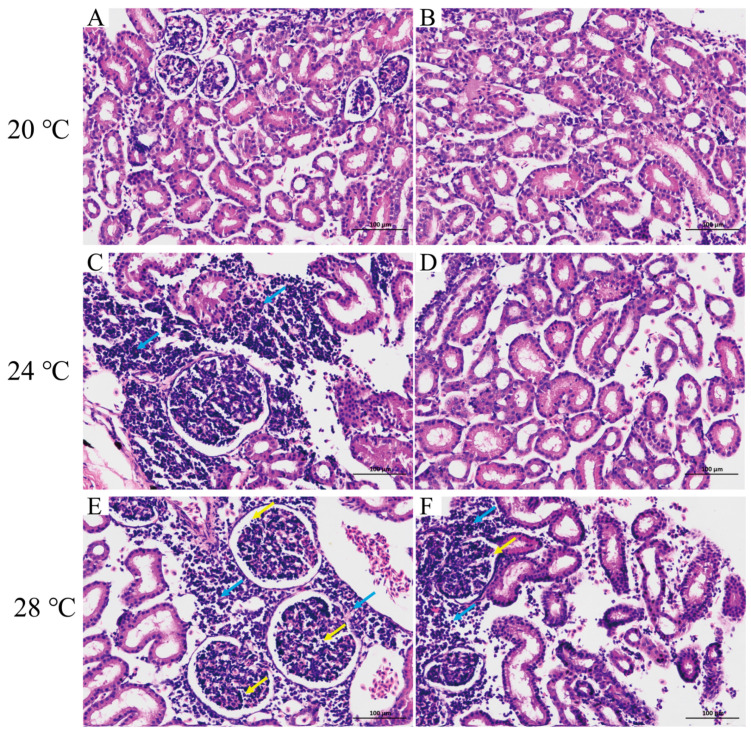
Histopathology of *A. baerii* kidney under heat stress. Control group ((**A**,**B**): 20 °C); heat stress groups ((**C**,**D**): 24 °C, (**E**,**F**): 28 °C). Yellow arrow: lymphocytic infiltration in the glomerulus; Blue arrow: lymphocyte infiltration in the interstitium.

The results of the histopathological observations revealed that heat stress caused damage to the kidney (Figure 2). In the control group, kidney corpuscles and tubule morphology were normal. Furthermore, the epithelial cells of the kidney tubules were rounded and full, with neatly and regularly arranged brush borders (Figure 2A,B). Heat stress led to substantial histopathological changes, specifically structural disturbances in the kidney and infiltration of lymphocytes compared to the control group (Figure 2C,F). Furthermore, with the increase in water temperature from 24 to 28 °C, heavy lymphocyte infiltration was observed (Figure 2E,F).

### 3.2. Apoptosis of Kidney Cells under Heat Stress

**Figure 3:**
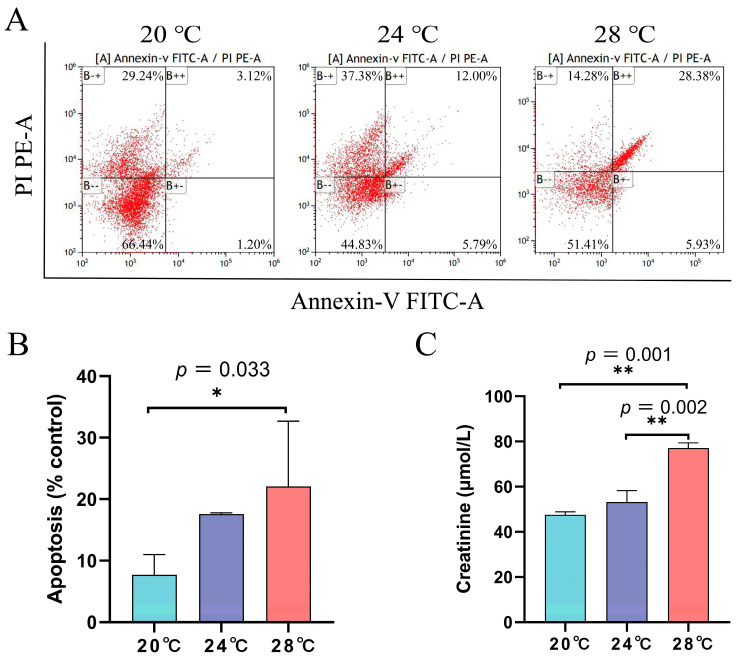
Heat stress led to apoptosis in kidney cells of *A. baerii* (**A**,**B**), along with an increase in plasma creatinine (**C**). Cell apoptosis was determined by flow cytometry after Annexin V-FITC/PI staining in kidney cells. The quantification data showed that apoptotic cell percentage was significantly higher after heat stress. * *p* < 0.05, ** *p* < 0.01, the detailed *p*-values are shown above.

As the intensity of heat stress increased, the rate of apoptosis also gradually increased, with the apoptosis rate at 28 °C being significantly higher than that of the control group (*p* = 0.033) (Figure 3A,B).

### 3.3. Changes in Plasma Creatinine under Heat Stress

Plasma creatinine levels were significantly increased in the 28 °C group compared to the control group (*p* = 0.001). In addition, plasma creatinine levels were also significantly higher in the 28 °C group than in the 24 °C group (*p* = 0.002) (Figure 3C).

### 3.4. Expression of Innate Immune Related Genes under Heat Stress

**Figure 4:**
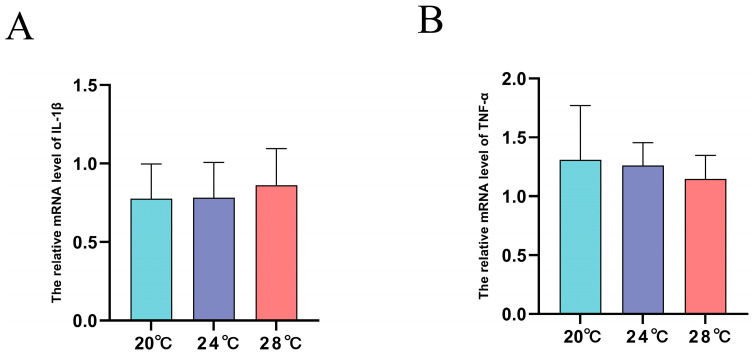
Relative mRNA expression level of *TNF-α* (**A**) and *IL-1β* (**B**) in the kidney of *A. baerii* (*n* = 3).

The expression of *IL-1β* mRNA was independent of temperature treatment (*p* > 0.05) (Figure 4A). In addition, the expression of *TNF-α* mRNA was also independent of temperature treatment (*p* > 0.05) (Figure 4B).

### 3.5. Expression of Heat Shock Protein-Related Genes under Heat Stress

**Figure 5:**
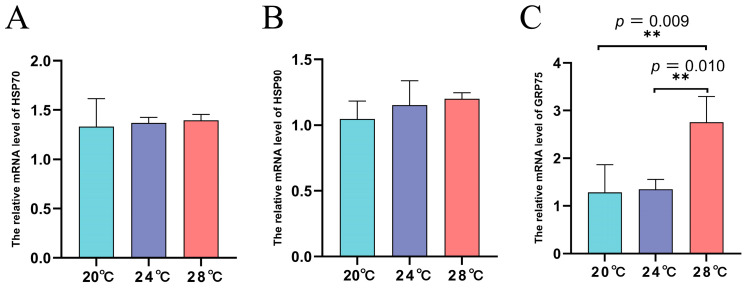
Relative mRNA expression levels of *HSP70* (**A**), *HSP90* (**B**), and *GRP75* (**C**) in kidney of *A. baerii* (*n* = 3). ** *p* < 0.01, the detailed *p*-values are shown above.

As temperature increased, expression levels of GRP75 mRNA in the 28 °C group significantly increased compared to the control group (*p* = 0.009) (Figure 5C). However, there was no significant difference in the expression levels of HSP70 and HSP90 mRNA in the heat stress group compared to control group (Figure 5A,B).

## 4. Discussion

In this study, we found that heat stress caused kidney structural disturbances and infiltration of lymphocytes in *A. baerii*. Severe damage occurred at 28 °C, with massive infiltration of inflammatory cells in the glomeruli and interstitium, suggesting that heat stress induces kidney injury in *A. baerii*, and the kidney damage is more severe with increasing intensity of heat stress. This is consistent with explorations on the kidney of *Notothenia coriiceps*. It can be observed that under the same heat stress treatment, the damage to the kidney is relatively mild compared with other tissues, which may be related to tissue specificity. In addition, studies on the kidneys of other fish have found that sesamin can protect against fluoride-induced oxidative stress and apoptosis in the kidney of carp (*Cyprinus carpio*) via the JNK signaling pathway [36], and herbicide paraquat dichloride causes necrosis of glomeruli and damage to the collecting duct in acute exposure [37].

Numerous studies have shown that apoptosis in fish cells occurs after exposure to heat stress [38]. Heat stress at the blastopore closure stage increases caspase3-mediated apoptosis in *P. lineatus* embryos [12]. One study revealed that the level of apoptosis in the Pikeperch (*Sander lucioperca*) liver tissue significantly increased with rising temperature [39]. In this study, we found that heat stress caused apoptosis in kidney cells, and the rate of apoptosis was significantly higher at 28 °C than in the control group. Therefore, the kidney of *A. baerii* showed severe damage at 28 °C.

Creatinine is the product of creatine metabolism in muscle tissue, and creatinine in fish is excreted through glomerular filtration. Creatinine accumulated in the blood when the function of glomerular filtration was impaired in large amounts [40]. Therefore, plasma creatinine content was an important indicator for the physiological function of kidneys in fish [41]. Heat stress resulted in impaired kidney function and significant increases in plasma creatinine and cortisol levels in *Scophthalmus maximus* [42]. In this study, it was found that plasma creatinine levels were significantly higher in the 28 °C heat stress group compared to the control group (*p* = 0.001). In addition, plasma creatinine levels were significantly higher in the 28 °C heat stress group than in the 24 °C stress group (*p* = 0.002). This indicated that the heat stress treatment at 24 °C caused little damage to kidney function, whereas the heat stress treatment at 28 °C caused severe damage to *A. baerii* kidney function. This is consistent with the study of plasma creatinine on *Pampus argenteus* [43].

Innate immunity is the earliest immune mechanism with a non-specific character which can be induced by the external environment and then respond by affecting the expression of cytokines such as *IL-1β* and *TNF-α* [44,45]. Therefore, the expression levels of cytokines such as *IL-1β* and *TNF-α* have been used as a tool to measure the immune response in fish [46]. Heat stress causes down-regulation of immune response genes in *Micropterus salmoides* and weakens disease resistance [18]. Heat stress also led to significant increases in the immune-related genes *TNF-α* and *TLR5* mRNA in *Oncorhynchus mykiss* kidneys [47]. Notably, there was no significant difference in the expression levels of *IL-1β* and *TNF-α* mRNA after heat stress compared with the control group, indicating that heat stress has a relatively minor effect on the immune response in the kidney of *A. baerii*. The lack of significant changes in *IL-1β* and *TNF-α* mRNA after heat stress may be due to the fact that the intensity of heat stress in this study was weak and did not elicit a dramatic immune response, or that the kidney was not a major target organ of heat stress [39].

When cells are exposed to various stressors, such as elevated temperature, hypoxia, and nutrient deficiency, the heat shock protein response can be transcriptionally regulated by heat shock factor-1 (HSF-1), which rapidly initiates the synthesis of heat shock proteins [48]. Based on sequence similarity and molecular size, heat shock proteins can be categorized into five major groups: HSP100, HSP90, HSP70, HSP60, and small heat shock proteins (sHSPs) [49]. Among the studies on fish heat shock proteins, the HSP70 and HSP90 families are the most intensively studied, and they are widespread and well-studied proteins [50,51]. HSPs play a critical role in coping with adverse environmental conditions, such as heat stress and hypoxic stress [52,53]. Multiple studies have demonstrated the key role of *HSP70* in the heat resistance of fish [54,55,56]. Moreover, HSP90 is involved in many cellular processes beyond protein folding, including DNA repair, development, and immune response, while GRP75 plays a cytoprotective role in maintaining cellular energy metabolism balance [57,58]. In this study, we investigated the mRNA expression levels of *HSP70, HSP90*, and *GRP75* in *A. baerii* following heat stress. Notably, there were no significant changes in the expression of *HSP90* and *HSP70* mRNA in the kidney after heat stress, which is inconsistent with previous studies of heat stress on gills of *A. baerii* [4]. Studies on *Triplophysa siluroides* also found that the *HSP70* and *HSP90* genes in the heat shock protein family were significantly up-regulated by heat stress at 28 °C [59]. Therefore, the reason for the absence of significant changes in the expression of *HSP90* and *HSP70* mRNA in the kidney of *A. baerii* after heat stress may be due to tissue specificity. The results indicate that the expression of *GRP75* mRNA in the kidney increased after heat stress, with the maximum expression level observed at 28 °C. This finding is consistent with previous studies conducted on gill, liver, and spleen [4]. This finding suggests that GRP75 may play a more significant role than other HSPs in mitigating heat stress in *A. baerii*. Therefore, our results suggest that *A. baerii* produces a robust stress response to the kidney of *A. baerii* during heat stress at 28 °C, leading to kidney inflammatory cell infiltration, apoptosis, and kidney dysfunction. Furthermore, our study reiterates the critical role of GRP75 in the heat stress response in fish.

## 5. Conclusions

Our results demonstrated that heat stress induced inflammatory cell infiltration in the kidney of *A. baerii*, along with a significant increase in the rate of apoptosis, a significant increase in creatinine levels, a marker of renal function, and triggered significant expression of heat shock protein *GRP75* mRNA to protect the kidney from heat stress injury. In addition, there was no significant change in kidney immune-related genes and the tissue lesions were less severe, suggesting that the kidneys are more heat-resistant. These findings contribute to the understanding of the coping strategies of the kidney of *A. baerii* for chronic heat stress.

## Figures and Tables

**Table 1:** Primers used for qPCR.

Gene	Primer Sequences (from 5′ to 3′)	Annealing Temperature (°C)
*TNF-α*	F: CAAGATTGTGGTGCCGAGGA	58.4
	R: GCAAGTCGCTCGATGTTGTG	
*IL-1β*	F: GAGAAGATGAAGAGACCGCA	60.0
	R: AGGATCACGTGCTCTTCATT	
*HSP90*	F: CGAGCTGTTGCGATACCAC	62.4
	R: CAACTTGGTCCTTGCTCTCAC	
*HSP70*	F: GCCAGCGGTGGATTTCACT	59.6
	R: TGCTATTGCTTATGGCTTGGAC	
*GRP75*	F: ACGGACTGAGTCAAGATGTC	60.0
	R: CTGTTTGCCTTCCATCACTG	
*β-actin*	F: TGGACGCCCAAGACATCAGG	59.6
	R: GGTGACAATGCCGTGCTCG	

## Data Availability

Data available on request due to privacy/ethical restrictions (the data that support the findings of this study are available on request from the corresponding author).
